# Objective Assessment of Physical Activity and Associated Contexts During High School Sport Practices

**DOI:** 10.3389/fspor.2021.548516

**Published:** 2021-07-09

**Authors:** Troy Carlton, Thomas L. McKenzie, Jason N. Bocarro, Michael Edwards, Jonathan Casper, Luis Suau, Michael A. Kanters

**Affiliations:** ^1^Catawba College, Salisbury, NC, United States; ^2^San Diego State University, San Diego, CA, United States; ^3^North Carolina State University, Raleigh, NC, United States; ^4^Shaw University, Raleigh, NC, United States

**Keywords:** youth sport, physical activity, objective measurement, practice context, sport practices

## Abstract

**Background:** Organized sports provide children and adolescents with opportunities to achieve recommended amounts of moderate to vigorous physical activity (MVPA), and schools are a primary setting for sports programs. The main aims of this study were to examine participant physical activity (PA) levels during the most popular high school sports in the United States and to assess the influences of practice contextual factors on PA levels.

**Methods:** Participant PA and its contexts were assessed during practices for the 10 most popular girls' and boys' high school sports in the United States. Data were obtained during 598 practice sessions in 12 schools in North Carolina using a validated direct observation instrument (System for Observing Fitness Instruction Time (SOFIT). A regression model was applied to understand the association between sport context and athletes' PA.

**Results:** Overall, athletes were observed engaging in MVPA 60% of practice time. MVPA varied among sports and levels were highly influenced by practice contexts. Among girls' sports, cross country and soccer practices provided the highest proportion of MVPA and MVPA percent during boys' practice sessions was highest during cross country and track and field. Practice contexts were associated with MVPA accrual with time allocated for gameplay and fitness activities associated with the highest levels of PA.

**Conclusions:** The results contribute to an understanding of which sports and how their practices are conducted facilitate increased PA. Findings indicate athletes accrue substantial amounts of PA during high school practices, but that it varies from sport to sport. As well, the context that characterizes sport practices is a significant determinant in how much PA occurs. Most sports that emphasized game simulation, fitness, and skill development drills had higher levels of MVPA. Given the length and frequency of practices and how the content is delivered, we can accurately predict how much PA athletes are likely to achieve during a given sport season. The findings from this study demonstrate that sport can make a useful, if not sufficient role in helping high school athletes reach recommended PA levels. PA engagement can be optimized by attending to the management of contexts surrounding the sports.

## Background

Participation in physical activity (PA) is associated with numerous health benefits in children and adolescents (Janssen and Leblanc, 2010) and current guidelines recommend children accrue 60 min of moderate-to-vigorous physical activity (MVPA) per day [U.S. Department of Health and Human Services (USDHHS), 2018]. Youth meeting this recommendation can be expected to improve their health and well-being in a number of different areas, including decreased cardiovascular disease risk factors (Carnethon et al., 2005), lower blood pressure (Nielsen and Andersen, 2003), weight management (Ness et al., 2007), increased bone density (Petit et al., 2002), muscular (Kriemler et al., 2011), and cardiovascular fitness (Fernhall and Agiovlasitis, 2008). Additionally, PA engagement during the early years has been shown to be strongly associated with the adoption of a more active lifestyle in adulthood (Ekblom-Bak et al., 2018).

Research reviews indicate children in the United States fall short of meeting recommended amounts of daily PA (Centers for Disease Control Prevention., 2018). For instance, only 22 percent of adolescents meet the national PA guidelines (CDC - Centers for Disease Control Prevention., 2018) and approximately one out of five high school aged youths are completely inactive (Physical Activity Council, 2019). Meanwhile, organized sports have been identified as an opportune strategy for adolescents to achieve recommended PA levels (Centers for Disease Control Prevention., 2018) and these are available in most communities in the form of private sports clubs, public recreational offerings, and school interscholastic athletics (Demetriou et al., 2017). In the United States, sports programs offered at schools are particularly important because children and adolescents spend a major proportion of their waking hours there. Middle and high schools, in particular, usually have well-trained/certified staff and facilities to support sport programs (Mears and Jago, 2016).

In the United States, 58 percent of youth, ages 6 to 17 years, participate in team sports or take sports lessons after school or on weekends [The Child Adolescent Health Measurement Initiative, 2017; U.S. Department of Health and Human Services (USDHHS), 2019]. Specifically within high schools, ~8 million students participate in sport programs annually (DeMartelaer and Theeboom, 2006) and these account for 54 percent of all eligible students. Given the large numbers participating in high school sport (and sport in general), coupled with the myriad of associated health and developmental benefits, sport has the potential to be a powerful health-promoting setting for adolescents, especially related to MVPA engagement (Wickel and Eisenmann, 2007).

Meanwhile the association between sport participation and PA remains unclear (Camire, 2014). Studies generally paint a positive picture of the role of sports participation on PA (Guagliano et al., 2013; Ball and Bice, 2015). For example, football and soccer participants were found to have more MVPA on sports days than non-sports days (Wickel and Eisenmann, 2007) and other studies found that the MVPA typically accrued on sport days was replaced by low-intensity, and sedentary activities on non-sport days (Machado-Rodrigues et al., 2012; Vella et al., 2013). These findings are consistent with other research indicating that adolescents are unlikely to compensate for missing MVPA when not engaging in sports (Dale et al., 2000) and suggest that sport has strong potential for increasing PA levels and reducing bouts of sedentary behavior (Kanters et al., 2015).

Some studies indicate MVPA during sport is dependent on organizational and environmental contexts such as facilities and coach behavior [National Federation of State High School Associations (NFHS), 2017]. Two studies, for example, have reported that adolescents spent up to 70 percent of their practice time engaged in sedentary or minimally active behavior (Wickel and Eisenmann, 2007; Kanters et al., 2015). Additionally, athletes have been reported to be physically active during only one third of the time during basketball and soccer practices (Guagliano et al., 2013) and soccer games (Sacheck et al., 2011). Nonetheless, the preponderance of research supports the notion that children and adolescents who play sports are more active than those who do not. With so many children participating in organized and adult-led physical activities (National Physical Activity Plan Alliance, 2018), the role that sports can play in adolescent development is undeniable. However, discussions on how organized sports serve as a method to facilitate PA and an active lifestyle are equivocal (Janssen and Leblanc, 2010; Demetriou et al., 2017; McKenzie, 2019).

Studies rarely examine how MVPA varies within the type and level of sport or within the contexts of sport practices (McKenzie, 2019). MVPA does not just “occur,” but is governed by the nature of the sport (e.g., rules, policies, and game strategies), space/facilities in which it occurs, and how coaches manage and structure practice time (Ball and Bice, 2015). High school sports in the U.S. are fragmented with little to no continuity from program to program (Camire, 2014). A downside to this lack of a cohesive interscholastic sports organization is the overabundance of untrained coaches (Seefeldt and Ewing, 1997; Conroy and Coatsworth, 2006) resulting in inefficient organization of sport practices that may be compromising associated outcomes like PA (Coakley, 2011; Bocarro et al., 2014; Côté and Vierimaa, 2014).

Moreover, large-scale studies using objective techniques to simultaneously examine PA and its contexts during high school sport practices are absent. Therefore, the primary aim of this study was to objectively measure the PA levels of high school female and male athletes during public high school sport practices and to assess the results based on sport type and associated with practice contexts, including how they were organized, structured, and delivered. The following research questions guided our inquiry: (a) how physically active are athletes during high school sports practices? (b) what are the variations in PA across different sports for boys and girls? and (c) how are practice contexts associated with MVPA accrual?

## Methods

Student athletes in 12 public high schools in the U.S. State of North Carolina (NC) participated. High school level was selected because it provides a setting in which the structure and delivery of sport is governed by a State level authority. Unlike club and community recreation sports which have fewer consistent rules regarding participation, season length, practice frequency and length and other factors, high school sports provide a more standardized setting that allows for greater generalizability across various settings and different sports. Schools were selected from a comprehensive list of all high schools in NC (*N* = 686) obtained from the North Carolina High School Athletic Association (NCHSAA). The lead author fielded interest from school officials via email and phone calls until a sizable index of potential sites was developed. The 12 schools (Table 1) were selected using a stratified random sample to ensure the representativeness that considered district-level per-pupil expenditure, percent of student body eligible for free/reduced lunch, percent of student body of racial minority, and geography (to prevent regional clustering) (U.S. Department of Education: Institutional of Education Sciences, 2017). Another consideration was school location, with schools having to be within 15 miles of a trained data collector. In most cases, data collectors were teachers and coaches already working on site so no travel was necessary. For participating, schools received a monetary gift card for the purchase of athletic supplies or equipment. Table 1 lists characteristics of the 12 participating schools.

**Table 1:** Characteristics of North Carolina High Schools and Students in the Study.

**School**	**Students #**	**Class**	**Title I school**	**% Free or reduced lunch**	**% Black**	**% Hispanic**	**% White**
1	1,689	4A	No	27.9	12.1	13.0	69.4
2	1,072	2A	Yes	35.7	11.8	8.7	75.7
3	933	2A	No	27.7	3.3	5.0	85.3
4	1,401	4A	No	36.9	28.3	5.1	61.2
5	1,851	4A	Yes	52.6	41.4	29.4	23.8
6	1,907	4A	Yes	39.1	26.2	17.6	46.8
7	1,512	4A	Yes	52.2	55.4	5.8	34.0
8	1,351	4A	No	32.6	0.9	5.6	89.3
9	1,398	3A	Yes	46.0	41.1	14.9	39.6
10	1,360	3A	Yes	63.2	14.9	41.8	38.1
11	1,519	4A	Yes	45.8	4.5	6.7	83.9
12	2,018	4A	No	53.6	58.9	16.2	17.9

Based on a national high school athletics participation survey [National Federation of State High School Associations (NFHS), 2017], the 10 most popular team sports for high school boys and girls were studied. Boys' sports included tennis, track and field, soccer, basketball, lacrosse, cross country, swimming, baseball, football, and wrestling. Girls' sports included tennis, track and field, soccer, basketball, lacrosse, cross country, swimming, softball, cheerleading, and volleyball. Sport practices were selected rather than competitions as athletes typically spend more time participating in them (often 5 days/week). As all team members (not just the most skilled) participate during them, practice sessions are more inclusive than competitions and coaches have more control over MVPA accrual (Kanters et al., 2015).

### Instrumentation and Procedures

The System for Observing Fitness Instruction Time (SOFIT) (McKenzie et al., 1991) was used to objectively assess the sport practices. This tool has been widely used to assess student PA and instructional contexts (e.g., how session time is spent and instructor behavior) nationally and internationally (McKenzie and Smith, 2017). SOFIT is based on momentary time-sampling techniques in which objective information on athlete PA levels and the practice contexts are assessed simultaneously. Immediately prior to the official start of each practice, data collectors randomly selected five participating athletes (four to observe and one as a backup). Observers were located to not interfere with instructional methods or materials, typically on the side of the field or court near the main instructional area. Once the practice started, data collectors observed the first athlete for 4 min by rotating between a 10 s observation interval that was followed by a 10 s record interval. Pacing was provided automatically by the observers' iPad; signaling when it was time to observe and record. During each observation interval, athlete PA level (i.e., lying down, sitting, standing [Sedentary]; walking [Moderate]; or Vigorous and practice context (i.e., how time was allocated—management, knowledge content, fitness, skill practice, gameplay, or other) were recorded.

Variable definitions were similar to those in the original SOFIT protocol (McKenzie et al., 1991), but the language was adjusted to reflect sport practices rather than PE lessons. Management time refers to time allocated to management of participants or the environment by engaging in non-sport tasks including transitions, break times, changing gear, and moving from one space to another. Knowledge content refers to time allocated to the acquisition of relevant knowledge such as technique, strategy, rules, and teaching from coaches. Fitness refers to activities with the major purpose of altering the physical state of the athlete in terms of cardiovascular endurance, strength, or flexibility. Skill practice refers to time allocated primarily to develop the sport-relevant skills. The primary characteristic is the targeting of a specific skill (e.g., dribbling a basketball) and the athlete repeating the drill in efforts to refine that skill. Game play refers to time allocated to applying the skills in a game-like setting including simulated competitions or scrimmages. Other refers to allocated time that does not fit into any of the previously identified categories (e.g., unorganized free play). After 12 iterations of 10 s observe/record intervals (i.e., 4 min), the remaining three athletes were observed in sequence. At the conclusion of the 12th iteration for the fourth athlete, the data collector observed the first athlete again and this process continued until practice ended.

To enter data, observers used the most recent version of iSOFIT® (v. 1.6.0), an interactive Apple® application compatible with iPad devices. The layout and main functions of iSOFIT® are similar to the original protocol and data collection follows the same procedures as SOFIT. The additional features enabled the streamlining of data processing, automated time-keeping, and secure data storage.

### Observer Training and Reliability Assessment

A total of 25 data collectors, typically teachers and coaches, were recruited from participating schools and were paid to participate in SOFIT observer training and data collection. Observers participated in a 2 h, face-to-face session with the project manager at their respective high schools using the latest SOFIT training materials (Mears and Jago, 2016; McKenzie and Smith, 2017); beginning in a classroom and then followed by interactive practice in the field. The data collectors were issued an iPad-mini™ device and trained in the use of the iSOFIT® app, and they were required to submit a 30 min practice observation to the project manager within 48 h of the training to demonstrate their comprehension of the protocols. The project manager reviewed the exported data from the practice sessions prior to clearing data collectors to begin observing practices.

During data collection, two trained data collectors simultaneously coded the same athletes independently during the practices and percent inter-observer agreement (IOA) was calculated using Cohen Kappa for observations of PA level and practice context (van der Mars, 1989). Each observer was assessed for reliability during a minimum of two practices. Overall, IOA for PA level was 0.73 with a rage from 0.62 to 0.98 and 0.73 for practice context with a range from 0.67 to 1.00. This indicates a high degree of reliability (McKenzie, 2019) but below the eighty percent level recommended for claiming excellent agreement (van der Mars, 1989). Yet, the consistency among observers was still within the acceptable range of other scholars who consider 0.70 and above as excellent agreement (Fleiss et al., 2003).

### Data Collection

Study methods were approved by the Institutional Review Board for Human Subjects Research at North Carolina State University prior to the start of the study. Sports were assigned to data collectors based on their availability and/or comfort with certain sports (e.g., some requested to observe only sports that matched their gender). Observers were asked to record at least 10 full practices for their respective sport by the end of the regular season. A total of 30 practice sessions were targeted to be observed for each of the 20 identified sports. Sports were categorized into one of three seasons: fall, winter, or spring. Each season had an official launch and end date although there were a few exceptions based on extenuating circumstances (e.g., weather). Each of the 20 high school sports were observed in their respective regular sport season (i.e., no postseason practice sessions).

### Analysis

Data were analyzed using the Statistical Package for Social Sciences (SPSS) Version 26.0 software. The unit of analyses was the sport practice session (*n* = 598 sessions). Sport type and practice context subcategories (i.e., management, fitness, knowledge, skill, game play, and other) served as the independent variables. The dependent variables were the subcategories of PA (i.e., lying down, sitting, standing [sedentary], walking/moderate, vigorous, and a combined category of moderate and vigorous physical activity [or MVPA]. The PA subcategory measures represent the proportion of practice observations/time coded as either sedentary, moderate, or vigorous activity. For each practice session, the proportion of practice time that the observed athletes spent in MVPA was automatically calculated by the iSOFIT® app.

Observed PA data were also converted to standard metabolic equivalents (METs) in order to compare estimated energy expended during practices (see Table 2). METs were calculated by multiplying participant's PA level with assigned energy expenditure values: 1.5 for sedentary, 3.0 for every moderate, and 6.0 for vigorous. These values are widely accepted and used for estimating energy expenditure based on observational data (Ainsworth et al., 2011; Edwards et al., 2014).

**Table 2:** High School Sports Ranked on Average METs PER PRACTICE.

**Sport type**	**Average METs**	**Std. Dev**.	**# Observations**
**Boys sports**			
Cross Country^*^^(*BTF, BBase, GCH, GSB, GTF*)^	4.84	1.75	2,794
Track and field^*^^(*BCC, BBase, GCH, GSB, GTF*)^	4.62	1.76	2,618
Swimming^*^^(*BW, GSW, BBase, GCH, GSB, GTF*)^	4.11	2.18	6,649
Wrestling^*^^(*GSW, BBase, GCH, GSB, GTF*)^	4.10	2.13	11,825
Soccer^*^^(*BSW, BW, GSW, BBase, GCH, GSB, GTF*)^	4.05	2.02	5,462
Lacrosse^*^^(*BBask, GV*)^	3.82	2.07	8,324
Basketball^*^^(*BF, BL, GV, BBase, GCH, GSB, GTF*)^	3.71	1.96	8,444
Football^*^^(*GL, BTN, BBask, BBase, GCH, GSB, GTF*)^	3.62	1.99	11,894
Tennis^*^^(*GBase, GTN, GL, BF, BBase, GCH, GSB, GTF*)^	3.61	2.05	3,580
Baseball^***^	3.02	1.98	3,338
**Girls sports**			
Cross country^*^^(*BBase, GCH, GSB, GTF*)^	4.35	1.98	2,187
Soccer^*^^(*BSO, GCC, BBase, GCH, GSB, GTF*)^	4.23	2.04	5,258
Swimming^*^^(*BW, BSW, BSO, BBase, GCH, GSB, GTF*)^	4.11	2.17	7,292
Basketball^*^^(*GTN, GL, BTN, BBase, GCH, GSB, GTF*)^	3.51	2.01	10,922
Volleyball^*^^(*BBask, BL, BBase, GCH, GSB, GTF*)^	3.89	2.08	6,888
Lacrosse^*^^(*GB, GTN, BTN, BF, BBase, GCH, GSB, GTF*)^	3.53	2.00	4,039
Tennis^*^^(*GB, GL, BTN, BBase, GCH, GSB, GTF*)^	3.46	1.98	5,501
Track and Field^***^	3.24	1.93	6,909
Softball^***^	2.76	1.72	4,930
Cheerleading^***^	2.41	1.64	6,432

*** *Where sports METs are significantly different for all sports and*

* *^subscripts^ Shows where sports METs are significantly different from specific sports at p < 0.05 using Dunn's post-hoc test. For the subscripts, G, girl followed by the sport and B boy followed by the sport*.

*METs, metabolic equivalent of athlete engagement*.

To test relationships between the independent variables and PA levels, we measured the categorical independent variables to test for differences in the means across the ordinal PA dependent variables, specifically for sport type. Chi-square tests for independence and association were performed to test associations between practice context and PA level Previous SOFIT studies have used one-way ANOVAs in order to determine whether percentages of time spent in MVPA differed or changed based on athlete PA and other contexts (Curtner-Smith et al., 2007; Cotton and O'Connor, 2013). Because our data did not meet all required assumptions for normality, Kruskal-Wallis tests were used as a non-parametric alternative. The Kruskal-Wallis test is a non-parametric test based on ranks. This test is also appropriate for ordinal level data as the primary dependent variable, and the advantage of using this type of test is that it can be used to calculate differences between two or more means (Ruxton and Beauchamp, 2008). In cases where statistically significant differences existed, pair-wise comparisons were made for each subgroup within that specific category using Dunn' *post-hoc* test. Level of significance for inferential tests was established at *p* < 0.05.

Ordinary least squares (OLS) regression was used to predict the association between practice context and observed mean levels of METs. Two dummy variables for Fall and Spring were created to control for seasons since is a categorical variable. Mean METs per observation was calculated by aggregating all METs scores from each interval within one observation and dividing METs total by the number of intervals. Practice was used as the unit of analysis.

## Results

Data collectors observed 2,408 total athletes during 696 h of practice time (i.e., during 598 varsity practices across the 20 sports) for a total of 125,218 individual observations. Mean practice length was 80 min, with high variability across sports (SD = 26.4 min). Overall, athletes engaged in vigorous activity 43% of practice time, moderate PA 17% of the time, and sedentary activity 40% of the time. For girls, cross country and soccer produced the highest proportion of MVPA with cheerleading and softball having the greatest amounts of sedentary behavior (Table 3). For boys, cross country and track had the highest amounts of MVPA and baseball had the greatest amounts of sedentary behavior (Table 3).

**Table 3:** Percent of practice time that athletes engaged in sedentary, moderate and vigorous physical activity.

**Girls sports**	**Sedentary (%)**	**Moderate (%)**	**Vigorous (%)**	**MVPA (%)**
Basketball	44.80	15.70	39.50	55.20
Cheerleading	71.50	12.40	16.00	28.40
Cross country	24.90	17.80	57.30	75.10
Lacrosse	40.10	22.30	37.60	59.90
Soccer	29.40	15.10	55.50	70.60
Softball	55.60	24.80	19.60	44.40
Swimming	38.60	5.00	56.40	61.40
Tennis	40.50	23.80	35.70	59.50
Track	45.70	23.50	30.70	54.20
Volleyball	36.50	15.50	48.00	63.50
**Boys sports**	**Sedentary (%)**	**Moderate (%)**	**Vigorous (%)**	**MVPA (%)**
Baseball	57.20	13.60	29.20	42.80
Basketball	32.90	26.90	40.20	67.10
Cross Country	13.50	18.30	68.20	86.50
Football	36.50	24.60	38.90	63.50
Lacrosse	37.20	17.00	45.80	62.80
Soccer	29.80	20.20	49.90	70.10
Swimming	39.10	4.30	56.60	60.90
Tennis	40.70	18.60	40.70	59.30
Track	12.50	27.40	60.10	87.50
Wrestling	36.50	8.40	55.10	63.50

Based on estimated MET values (Table 2), boys cross country produced the highest average MET value followed by boys' track and field and girls' cross country. Sports with the lowest MET levels were girls' cheerleading, girls' softball, boys' baseball, girls' track and field, and girls' tennis. Overall, boys expended more energy during practices (m = 3.89) than girls (m = 3.51) (*F* = 215.72, *p* < 0.001).

Separate Kruskal-Wallis tests revealed there were significant differences in the PA engagement of athletes by sport type, and practice contexts (*p* < 0.001). Overall, a majority of practice time was allocated to skill development activities (36%), defined as activities repeated for the purposes of targeting a specific skill. Fitness was the next most common context (19%), followed by game play (15%), knowledge (14%), management (11%), and other (4%) (Table 4). The girls' sports with the highest amount of practice time allocated for game play were soccer, softball, and tennis. Boys' sports with the highest amount of game play were tennis, cross country, and basketball. Sports with the greatest proportion of practice time for fitness development were cross country, swimming, and track and field for both genders (Table 5).

**Table 4:** Overall percent of time allocated to practice contexts.

**Context**	**% of observed practice time**
Fitness	19.0%
Game play	14.8%
Knowledge content	13.60%
Management	11.0%
Skill practice	35.9%
Other	5.80%

**Table 5:** Percent of practice time for sports by gender.

**Girls sports**	**Fitness (%)**	**Game play (%)**	**Knowledge (%)**	**Management (%)**	**Skill practice (%)**	**Other (%)**
Basketball	7.40	12.60	26.90	8.50	44.20	0.50
Cheerleading	18.00	8.00	22.50	9.70	34.80	7.00
Cross country	33.80	12.80	8.20	15.70	14.50	15.00
Lacrosse	17.70	21.70	12.40	6.70	38.90	2.60
Soccer	14.90	30.90	16.60	5.10	26.70	5.70
Softball	10.20	22.10	12.60	6.80	44.30	4.00
Swimming	30.80	1.30	9.20	22.70	27.80	8.30
Tennis	13.50	21.80	12.90	4.30	36.90	10.60
Track	31.10	2.70	10.00	30.30	21.40	4.40
Volleyball	11.60	17.90	9.70	11.20	48.40	0.90
**Boys sports**	**Fitness (%)**	**Game play (%)**	**Knowledge (%)**	**Management (%)**	**Skill practice (%)**	**Other (%)**
Baseball	17.10	22.90	14.10	3.00	41.30	1.60
Basketball	4.60	22.30	10.30	13.90	42.40	6.60
Cross Country	40.20	32.80	9.80	11.00	4.60	1.50
Football	9.70	14.30	11.30	11.00	53.00	0.40
Lacrosse	17.40	18.90	14.00	17.10	30.20	2.40
Soccer	27.30	20.20	12.90	6.90	27.40	5.20
Swimming	39.30	2.20	11.60	14.50	29.00	3.30
Tennis	9.50	40.30	12.90	4.70	31.70	0.90
Track	31.40	15.40	14.00	8.90	30.00	0.20
Wrestling	28.90	5.00	14.40	3.50	46.30	1.90

Mean METs were calculated at the observational level for each of the six practice contexts. A chi-square test of independence revealed a statistically significant association between practice context and PA level (χ^2^ = 42560.39, *p* < 0.001) and a statistically significant difference in METs were observed between the contexts, *F*_(6, 123499)_ = 8793.78, *p* < 0.001).

Practice time allocated to game play (4.67 MET average) was associated with higher levels of MVPA. Practice time allocated for fitness activities (4.54) and skill practice (4.28) were both also associated with mean PA levels above 4.00 METs. Practice time allocated for other activities (2.18), management (2.11), and knowledge content (1.71) had reduced MET levels with a higher likelihood of sedentary activity.

### Practice Context-Level Model

An ordinary least squares (OLS) regression analysis was conducted to establish whether differences in levels of observed METs (outcome variable) existed across practice context factors (Table 6). The variables were tested for multicollinearity and the presence of outliers. We also examine a potential effect of seasons (e.g., Fall, Winter, and Spring) on and METs levels.

**Table 6:** Ordinary Least Squares Regression Analysis for Mets.

**Model**	***B***	**Beta**	**S.E**.	***p***
Constant	4.210	–	0.177	0.000
Fall	−0.326	−0.152	0.093	0.001
Spring	−0.399	−0.184	0.096	0.000
Fitness %	0.006	0.134	0.002	0.002
Game play %	0.007	0.155	0.002	0.000
Knowledge content %	−0.016	−0.245	0.003	0.000
Management %	−0.019	−0.232	0.003	0.000
Other %	−0.017	−0.162	0.004	0.000
*N*		598		
R2		0.18		

For our model, the amount of time spent in knowledge, management, and other practice context factors significantly reduced mean METs. However, time in fitness related activities and game play significantly increased mean METs in comparison to skill development activities (Table 6). Seasons or time of year were included in the model and when compared to Winter, Fall, and Spring significantly reduced mean levels of METs.

Since METs were calculated from levels of physical activity, a cross-tabulation between time of year and levels of physical activity showed that Fall sports had 39.1% of participants sedentary, 19.7% moderate and 41.2% vigorous. Spring sports had a similar pattern with 40.8% sedentary, 19.8% moderate and 39.4% in vigorous activity. Winter sports, however, had about the same sedentary levels (38.6%) but lower moderate (12.6%) and higher vigorous levels of participation with 48.8%.

## Discussion

The primary purpose of this study was to examine sport practice participation and its contexts on the PA of high school athletes. More specifically, it assessed the association between PA output by sport participants and the practice activities in 10 different sports for both male and female athletes. The study aimed to provide insight into the differences in PA between particular sports and determine what contextual factors are most influential for MVPA during sport practices.

This study is the first to provide a detailed account of a large cross-section of high school sport practices using SOFIT, an observational tool that simultaneously examines athlete PA and practice contextual information. Although there are no specific recommendations for the amount of time to be spent in MVPA for youth sports, there are guidelines for PE classes, which recommend students engage in MVPA at least 50 percent of lesson time (U.S. Department of Health and Human Services (USDHHS), 2012). The results from our study indicate that across all sports, 60 percent of practice time was spent in MVPA, exceeding the PE benchmark. Study findings can be summarized within three categories that contribute to our understanding of PA in youth sport settings: (1) gender differences; (2) sport type; and (3) the influence of practice context.

First, consistent with previous work comparing girls and boys in after-school settings (Bocarro et al., 2014), boys were more physically active during practices than girls. As Leek et al. (2011) found in baseball/softball practices, only two percent of girls obtained 60 or more minutes of MVPA compared to about 32 percent of boys (Leek et al., 2011).

Gender inequality in PA engagement is a persistent concern, magnified by lower PA among girls. Furthermore, there is a clear trend of decreasing PA levels with aging and with the disparity between girls and boys widening (Bailey et al., 2004). For example, one study estimated that PA declined during high school by 7.4 percent for girls and only 2.7 percent for boys (Sallis, 1993). Another study suggested female adolescents were ~20 percent less active than their male peers (Booth, 1997). The picture for sports participation is more positive in some (but not all) developed countries. In the U.S., there has been an increase in the number of girls participating in sports over recent decades (Coakley, 2011) but many still do not participate because of limited opportunities, structural barriers, and gender stereotypes and their participation rates do not match those of boys (Bailey et al., 2004). As such, girls have been identified as a high priority group for PA promotion (Biddle et al., 2014). Gender role stereotyping exists in sports, and coaches and parents play an influential role in both the selection of which sports to play and approaches to practice and play (Coakley, 2011). This study's findings supports the notion that coaches may frame practices differently based on athlete gender. However, further study of coach behavior, practice context, and subsequent physical activity of boys and girls sports is warranted.

Second, the data indicate that athletes were more active during practices in running-centric sports than other sports. This finding supports the conventional wisdom that athletes in sports such as cross country and soccer achieve more MVPA than sports characterized by more bouts of inactivity like cheerleading and baseball (Leek et al., 2011). Also, the sports with a lower proportion of MVPA during practice time were those that require athletes to be proficient in technical skills. For example, baseball and football may require increased practice time allocated to skill building activities, teaching tactical strategy, or other inactive instruction. While sports that require extensive aerobic capacity, such as swimming and track, are likely to allocate more practice to MVPA activities. As well, team (vs, “individual”) sports require practice time be allocated to developing collaboration/unity/tactics among athletes (especially those that demand team strategies) would likely allocate more practice time dedicated to strategy and other more sedentary activities.

Third, practice context factors highly influenced the amount and level of PA. Sports with more time allocated to game play, fitness, and skill practice activities were associated with increased MVPA (80, 79, and 72% of context time, respectively). Conversely, MVPA was lowest during knowledge and management contexts (10 and 37% of the time, respectively). Meanwhile, it is important to consider there are other goals of participating in sport (e.g., social and cognitive development) than PA and reaching these may require athletes to be stationary (Jones et al., 2016). Thus, practices should balance allocated time to facilitate participants' achievement of a full range of intended benefits, including adequate bouts of MVPA.

An unexpected finding was how close fitness and game play were in their production of PA. Athletes in fitness activities during practice would be expected to be physically active, but game play is not so intuitive. As previously indicated, a characteristic of the game play code was practice activity “generally performed without major intervention from the coach” that mimics a real game/match/meet. Hence, while scrimmaging in a game-like simulation, with little contact with the coach, athletes were more likely to be physically active during fitness and game play than during any other practice context codes.

While a limited body of literature has examined youth sport program delivery, the few that exist confirm that the context of youths' sport programs can play a significant role in the sport outcomes experienced (Fraser-Thomas et al., 2005; Leek et al., 2011; Sacheck et al., 2011; Guagliano et al., 2014, 2015; Kanters et al., 2015; Ridley et al., 2018; Schlechter et al., 2018). Recent research has supported that high school athletes spend <50 percent of practice time engaged in MVPA during sports practices (Schlechter et al., 2017). Although social influencers, organizational capacities, and program features may impact the ability of youth sport programs to facilitate healthy outcomes (Doherty and Cousens, 2013), empirical research to back up these claims is limited. As a result, there is a large body of literature linking sport with a variety of physical, social, and psychological outcomes but little insight on the practical implications for understanding this process.

The benefits of sports are much more likely to occur when quality, intentional programming is provided (President's Council on Sports, 2019). Quality sports programs typically focus on individual and team development through skill building while recognizing the value of a well-balanced practice that may include sedentary time. Sports-based development programs may provide youth with a substantial amount of PA (Perkins and Borden, 2006). In fact, the recent National Youth Sports Strategy report focuses on the benefits of sports participation and strategies to create quality sports programming that will increase the likelihood of youths obtaining these benefits [U.S. Department of Health and Human Services (USDHHS), 2019].

Although large amounts of sedentary activity were found during knowledge transfer and management activities, these components of a practice are not necessarily negative when considering the holistic development of an athlete. Physical inactivity or sedentary behavior is independent of PA and is also multi-dimensional. Although the biomedical view of PA is critical of excessive inactivity, many sedentary behaviors are highly valued such as school, studying, or reading and have benefits for young people. PA and inactivity are independent behaviors performed in a societal context, and both have high value in a sport practice. There is a need for better understanding of the meanings attached to and motivations for PA and sedentary behaviors by adolescents, particularly at the high school age. Most U.S. high schools (91%) offer students opportunities to participate in at least one interscholastic sport per year (Lee et al., 2007).

Time of year or season showed a positive association with levels of physical activity. While most seasons showed similar patterns of physical activity level distribution, it is worth noting that winter showed lower levels of moderate but the highest levels of vigorous physical activity of all seasons. However, it is important to note that while time of year may influence the level of PA among sport participants and that activity levels in general are higher when the weather is warmer, high school sports are intentionally scheduled to minimize this effect. For example, outdoor sports like football and baseball are scheduled in the early Fall and late Spring, while indoor sports like basketball and swimming occur during the winter. Therefore, the finding that higher amounts of vigorous activity were reported during the Winter season is likely due to the indoor sports that occur during that time (e.g., basketball and swimming). Furthermore, the intent of this study was to examine the activity levels of high school sport participants during practices and to identify the influence of practice context factors. While seasonal variations may impact activity levels at times (e.g., during significant inclement weather) the overall impact on participant activity across the various high school sports is likely very low.

The current study indicates that athletes were generally very active during practices, however, some sports (e.g., baseball and cheerleading) could be enhanced by implementing strategies to increase PA during all practice contexts. For example, splitting the team into different subgroups or establishing stations for the same activity could reduce sedentary time spent standing in line to participate (Kanters et al., 2015). Also, coaches could minimize time spent doing management tasks (e.g., transition periods and equipment setup). Providing coaches with more support, education, and strategies on how to design practices to maximize PA outcomes has been recommended (Schlechter et al., 2018) and are highly encouraged by the current study. Thus, there is clearly an opportunity to optimize MVPA levels and reduce inactivity and light PA during sport practices by modifying the activities themselves as well as the logistics of how to maximize the playing space.

There were several limitations to the study. Data were collected during only one traditional American high school academic year and was restricted to North Carolina high schools only. Not all high school sports were included (e.g., ice hockey and golf) and only 12 representative schools were included, limiting the power and generalizability of our analysis. Additional studies are needed to capture a larger cross section of schools in different regions of the country. As well, conclusions on PA in sports should recognize that participation is influenced by diverse biological, behavioral, cultural, and psycho-social factors–these were investigated in this study.

As well, studies are needed to examine how the built environment influences practice management and subsequent activity levels of participants. For example, low levels of PA during a swimming practice study was partly explained by the small size of the pool (Warburton and Woods, 1996). This brings about an important question when addressing the delivery of sport in school settings: Is there enough space for coaches to support large bouts of MVPA during practice time? Theoretically, more people in a small space yields less PA than fewer people in a large area, regardless of the practice context. However, highly structured activities delivered by trained coaches in confined spaces have demonstrated comparable PA levels to the same activities delivered in larger spaces (Kanters et al., 2015).

Despite these limitations, this study used objective measures to rigorously describe PA levels accrued during practices in school sports with the highest participation rates in the U.S. [National Federation of State High School Associations (NFHS), 2017]. The results add to the understanding of the contribution of sports to PA during practices. Additionally, the context that characterizes sport practices is significant in determining how much PA occurs. Sports that allocate emphasis on game simulation, fitness, and skill development can be expected to have higher levels of MVPA. Based on how practices are delivered, it is possible to predict how much PA athletes can expect to achieve within a given sport season assuming length and frequency of practices are recorded.

Sports have unique characteristics that suggest it can play a valuable role in the promotion of PA, especially during the teenage years. The findings from this study demonstrate that sport can make a useful, if not sufficient role in helping high school athletes reach recommended PA levels. The effect this has on later PA is not yet known, but a case can be made for a generally positive influence of sporting context.

## Data Availability Statement

The datasets presented in this article are not readily available because the funding agency has prohibited the sharing of data from this project. Requests to access the datasets should be directed to Michael A. Kanters, mkanters@ncsu.edu.

## Ethics Statement

The studies involving human participants were reviewed and approved by NC State University Institutional Review Board. Written informed consent to participate in this study was provided by the participants' legal guardian/next of kin.

## Author Contributions

MK project principal investigator and coordinated overall management of the study. TC coordinated all data collection for the project and lead writing of this article. JB assisted with data collection and contributed to writing of background and discussion sections. ME lead analyst for analysis of data and lead writing of results section for the article. JC assisted with management of data and analysis and writing of results section. TM developed measurement method used and assisted with overall manuscript writing. LS assisted with data collection and inter reliability assessment and overall data manager for project. All authors contributed to the article and approved the submitted version.

## Conflict of Interest

The authors declare that the research was conducted in the absence of any commercial or financial relationships that could be construed as a potential conflict of interest.

## References

[B1] Ainsworth B. E. Haskell W. L. Herrmann S. D. Meckes N.. (2011). 2011 compendium of physical activities: a second update of codes and MET values. Med. Sci. Sports Exerc. 1575–1581. 10.1249/MSS.0b013e31821ece12 21681120

[B2] Bailey R. P. Wellard I. Dismore H. (2004). Girls' Participation in Physical Activities and Sports: Benefits, Patterns, Influences and Ways Forward. Geneva: World Health Organization.

[B3] Ball J. W. Bice M. R. (2015). Adult BMI and physical activity: retrospective evaluation of high school sport and recreation participation. Recreat. Sports J. 39, 144–156. 10.1123/rsj.2015-0065

[B4] Biddle S. J. Braithwaite R. Pearson N. (2014). The effectiveness of interventions to increase physical activity among young girls: a meta-analysis. Prev. Med. 62, 119–131. 10.1016/j.ypmed.2014.02.009 24530611

[B5] Bocarro J. N. Kanters M. A. Edwards M. B. Casper J. M. McKenzie T. L. (2014). Prioritizing school intramural and interscholastic programs based on observed physical activity. Am. J. Health Promot. 28, S65–S71. 10.4278/ajhp.130430-QUAN-205 24380468

[B6] Booth M. (1997). The NSW schools fitness and physical activity survey. 1997. NSW Public Health Bull. 8, 35–36. 10.1071/NB97016 9732115

[B7] Camire M. (2014). Youth development in North American high school sport: review and recommendations. Quest 66, 495–511. 10.1080/00336297.2014.952448

[B8] Carnethon M. R. Gulati M. Greenland P. (2005). Prevalence and cardiovascular disease correlates of low cardiorespiratory fitness in adolescents and adults. JAMA 294, 2981–2988. 10.1001/jama.294.23.2981 16414945

[B9] CDC - Centers for Disease Control Prevention. (2018). Youth Risk Behavior Surveillance – United States. 2017. Morbidity and Mortality Weekly Report. 67. Atlanta, GA: United States Department of Health and Human Services. Retrieved from: https://www.cdc.gov/healthyyouth/data/yrbs/.

[B10] Centers for Disease Control and Prevention. (2018). Youth Risk Behavior Surveillance – United States, 2017. Morbidity and Mortality Weekly Report, Atlanta, GA. 10.15585/mmwr.ss6708a1 PMC6002027 29902162

[B11] Coakley J. (2011). Youth sports: what counts as positive development? J. Sport Soc. Issues 35, 306–324. 10.1177/0193723511417311

[B12] Conroy D. E. Coatsworth J. D. (2006). Coach training as a strategy for promoting youth social development. Sport Psychol. 20, 281–292. 10.1123/tsp.20.2.128

[B13] Côté J. Vierimaa M. (2014). The developmental model of sport participation: 15 years after its first conceptualization. Sci. Sports 29, S63–S69. 10.1016/j.scispo.2014.08.133

[B14] Cotton W. O'Connor D. (2013). “The effect of a training evaluation tool on youth coaches,” in Science and Football VII: The Proceedings of the Seventh World Congress on Science and Football, eds H. Nunome, B. Drust, and B. Dawson (New York, NY: Routledge), 385–390.

[B15] Curtner-Smith M. D. Sofo S. Chouinard J. Wallace S. (2007). Health-promoting physical activity and extra-curricular sport. Eur. Phys. Educ. Rev. 13, 131–144. 10.1177/1356336X07076871

[B16] Dale D. Corbin C. B. Dale K. S. (2000). Restricting opportunities to be active during school time: do children compensate by increasing physical activity levels after school? Res. Q. Exerc. Sport. 71, 240–248. 10.1080/02701367.2000.10608904 10999261

[B17] DeMartelaer K. Theeboom M. (2006). “Physical education and youth sport,” in The Handbook of Physical Education, eds D. Kirk, D. Macdonald, and M. O'Sullivan (Thousand Oaks, CA; London: Sage).

[B18] Demetriou Y. Gillison F. McKenzie T. L. (2017). After-school physical activity interventions on child and adolescent physical activity and health: a review of reviews. Adv. Phys. Educ. 7, 191–215. 10.4236/ape.2017.72017

[B19] Doherty A. Cousens L. (2013). Introduction to the special issue on community sport. J. Sport Manag. 27, 419–421. 10.1123/jsm.27.6.419

[B20] Edwards M. B. Kanters M. A. Bocarro J. N. (2014). Policy changes to implement intramural sports in North Carolina middle schools: simulated effects on sports participation rates and physical activity intensity. 2008-2009. Prev. Chronic Dis. 11:130195. 10.5888/pcd11.130195 24433623 PMC3894929

[B21] Ekblom-Bak E. Ekblom Ö. Andersson G. Wallin P. Ekblom B. (2018). Physical education and leisure-time physical activity in youth are both important for adulthood activity, physical performance, and health. J. Phys. Activity Health 15, 661–670. 10.1123/jpah.2017-0083 29706117

[B22] Fernhall B. Agiovlasitis S. (2008). Arterial function in youth: window into cardiovascular risk. J. Appl. Physiol. 105, 325–333. 10.1152/japplphysiol.00001.2008 18450990

[B23] Fleiss L. Levin B. Paik M. C. (2003). “The measurement of interrater agreement,” in Statistical Methods for Rates and Proportions, 3rd Edn, eds J.L. Fleiss, B. Levin, and M. Cho Paik (Hoboken, NJ: John Wiley and Sons, Inc.), 598–626.

[B24] Fraser-Thomas J. L. Côté J. E. Deakin J. (2005). Youth sport programs: an avenue to foster positive youth development. Phys. Educ. Sport Pedagogy 10, 19–40. 10.1080/1740898042000334890

[B25] Guagliano J. Rosenkranz R. Kolt G. (2013). Girls' physical activity levels during organized sports in Australia. Med. Sci. Sports Exerc. 45, 116–122. 10.1249/MSS.0b013e31826a0a73 22843107

[B26] Guagliano J. M. Lonsdale C. Kolt G. S. Rosenkranz R. R. (2014). Increasing girls' physical activity during an organized youth sport basketball program: a randomized controlled trial protocol. BMC Public Health 14:383. 10.1186/1471-2458-14-383 24751173 PMC4098641

[B27] Guagliano J. M. Lonsdale C. Kolt G. S. Rosenkranz R. R. George E. S. (2015). Increasing girls' physical activity during a short-term organized youth sport basketball program: a randomized controlled trial. J. Sci. Med. Sport 18, 412–417. 10.1016/j.jsams.2015.01.014 25728339

[B28] Janssen I. Leblanc A. G. (2010). Systematic review of the health benefits of physical activity and fitness in school-aged children and youth. Int. J. Behav. Nutr. Phys. Activity 7:40. 10.1186/1479-5868-7-40 20459784 PMC2885312

[B29] Jones G. J. Edwards M. B. Bocarro J. N. Bunds K. S. Smith J. W. (2016). An integrative review of sport-based youth development literature. Sport Soc. 20, 161–179. 10.1080/17430437.2015.1124569

[B30] Kanters M. Edwards M. Casper T. McKenzie T. Bocarro J. Carlton T.. (2019). Physical Activity Report. Available online at: https://healthysportindex.com/report/physical-activity/

[B31] Kanters M. McKenzie T. Edwards M. Bocarro J. Mahar M. Hodge C. (2015). Youth sport practice model gets more kids active with more time practicing skills. Retos 28, 173–177. 10.47197/retos.v0i28.34951

[B32] Kriemler S. Meyer U. Martin E. van Sluijs E. M. Andersen L. B. Martin B. W. (2011). Effect of school-based interventions on physical activity and fitness in children and adolescents: a review of reviews and systematic update. Br. J. Sports Med. 45, 886–895. 10.1136/bjsports-2011-090186 21836176 PMC3841814

[B33] Lee S. M. Burgeson C. R. Fulton J. E. Spain C. G. (2007). Physical education and physical activity: results from the school health policies and programs study 2006. J. Sch. Health 77, 435–463. 10.1111/j.1746-1561.2007.00229.x 17908102

[B34] Leek D. Carlson J. A. Cain K. L. Henrichon S. Rosenberg D. Patrick K.. (2011). Physical activity during youth sports practices. Arch. Pediatr. Adolesc. Med. 165, 294–299. 10.1001/archpediatrics.2010.252 21135319

[B35] Machado-Rodrigues A. M. Coelho-e-Silva M. J. Mota J. Santos R. M. Cumming S. Malina R. M. (2012). Physical activity and energy expenditure in adolescent male sport participants and non-participants aged 13-16 years. J. Phys. Activity Health 9, 626–633. 10.1123/jpah.9.5.626 21953445

[B36] McKenzie T. L. (2019). Physical activity within school context: the bigger bang theory. Kinesiol. Rev. 8, 48–53. 10.1123/kr.2018-0057

[B37] McKenzie T. L. Sallis J. F. Nader P. R. (1991). SOFIT: system for observing fitness instruction time. J. Teach. Phys. Educ. 11, 195–205. 10.1123/jtpe.11.2.19522485071

[B38] McKenzie T. L. Smith N. J. (2017). Studies of physical education in the United States using SOFIT: a review. Res. Q. Exerc. Sport 88, 492–502. 10.1080/02701367.2017.1376028 29064778

[B39] Mears R. Jago R. (2016). Effectiveness of after-school interventions at increasing moderate-to-vigorous physical activity levels in 5- to 18-year olds: a systematic review and meta-analysis. Br. J. Sports Med. 50, 1315–1324. 10.1136/bjsports-2015-094976 27222308

[B40] National Federation of State High School Associations (NFHS) (2017). 2017 Handbook. Retrieved from: http://www.nfhs.org/ParticipationStatistics/PDF/2016-17_Participation_Survey_Results.pdf

[B41] National Physical Activity Plan Alliance (2018). The 2018 United States Report Card on Physical Activity for Children and Youth. Washington, DC.

[B42] Ness A. R. Leary S. D. Mattocks C. Blair S. N. Reilly J. J. Wells J.. (2007). Objectively measured physical activity and fat mass in a large cohort of children. PLoS Med. 4:e97. 10.1371/journal.pmed.0040097 17388663 PMC1831734

[B43] Nielsen G. A. Andersen L. B. (2003). The association between high blood pressure, physical fitness, and body mass index in adolescents. Prev. Med. 36, 229–234. 10.1016/S0091-7435(02)00017-8 12590998

[B44] Perkins D. F. Borden L. M. (2006). “Competitive sports pressure and out-of-school learning experiences,” in Adolescent Mental Health, eds F. A. Villarruel and T. Luster (Westport, CA: Greenwood Press), 239–256.

[B45] Petit M. A. McKay H. A. MacKelvie K. J. Heinonen A. Khan K. M. Beck T. J. (2002). A randomized school-based jumping intervention confers site and maturity-specific benefits on bone structural properties in girls: a hip structural analysis study. J. Bone Mineral Res. 17, 363–372. 10.1359/jbmr.2002.17.3.363 11874228

[B46] Physical Activity Council (2019). 2019 Physical Activity Council's Overview Report on U.S. Participation. Jupiter, FL: Sports Marketing Surveys USA.

[B47] President's Council on Sports Fitness and Nutrition. (2019). Physical Activity Guidelines for Americans Midcourse Report: Strategies to Increase Physical Activity Among Youth, 1–48.

[B48] Ridley K. Zabeen S. Lunnay B. K. (2018). Children's physical activity levels during organized sports practices. J. Sci. Med. Sport 21, 930–934. 10.1016/j.jsams.2018.01.019 29452749

[B49] Ruxton G. D. Beauchamp G. (2008). Some suggestions about appropriate use of the Kruskal-Wallis test. Anim. Behav. 76, 1083–1087. 10.1016/j.anbehav.2008.04.011

[B50] Sacheck J. Nelson T. Ficker L. Kafka T. Kuder J. Economos C. (2011). Physical activity during soccer and its contribution to physical activity recommendations in normal weight and overweight children. Pediatr. Exerc. Sci. 23, 281–292. 10.1123/pes.23.2.281 21633140

[B51] Sallis J. F. (1993). Epidemiology of physical activity and fitness in children and adolescents. Crit. Rev. Food Sci. Nutr. 33, 403–408. 10.1080/10408399309527639 8357503

[B52] Schlechter C. R. Guagliano J. M. Rosenkranz R. R. Milliken G. A. Dzewaltowski D. A. (2018). Physical activity patterns across time-segmented youth sport flag football practice. BMC Public Health 18:226. 10.1186/s12889-018-5108-3 29422038 PMC5806374

[B53] Schlechter C. R. Rosenkranz R. R. Milliken G. A. Dzewaltowski D. A. (2017). Physical activity levels during youth sport practice: does coach training or experience have an influence? J. Sports Sci. 35, 22–28. 10.1080/02640414.2016.1154593 26930302

[B54] Seefeldt V. D. Ewing M. E. (1997). Youth sports in American: an overview. President's Council Phys. Fitness Sports Res. Digest 2, 1–14.

[B55] The Child Adolescent Health Measurement Initiative (2017). 2017 National Survey of Children's Health (NSCH) Data Query. Data Resource Center for Child and Adolescent Health (databased online). Baltimore, MD: Johns Hopkins Bloomberg School of Public Health. Available online at: www.childhealthdata.org (access October 10, 2019).

[B56] U.S. Department of Education: Institutional of Education Sciences (2017). Data and Tools. National Center for Education Statistics. Retrieved from: https://nces.ed.gov/

[B57] U.S. Department of Health Human Services (USDHHS) (2012). Physical Activity Guidelines for American's Midcourse Report: Strategies to Increase Physical Activity Among Youth. Washington, DC. Retrieved from: https://health.gov/paguidelines/2008/midcourse/pag-mid-course-report-final.pdf

[B58] U.S. Department of Health Human Services (USDHHS) (2018). Physical Activity Guidelines for Americans, 2nd Edn. Washington, DC. Retrieved from: https://health.gov/paguidelines/second-edition/

[B59] U.S. Department of Health Human Services (USDHHS) (2019). National Youth Sports Strategy. Washington, DC. Retrieved from: https://health.gov/paguidelines/youth-sports-strategy/pdf/National_Youth_Sports_Strategy.pdf

[B60] van der Mars H. (1989). “Observer reliability: issues and procedures,” in Analyzing Physical Education and Sport Instruction, 2nd Edn, eds P. W. Darst, D. B. Zakrajsek, and V. H. Mancini (Champaign, IL: Human Kinetics), 53–80.

[B61] Vella S. Cliff D. P. Okely A. D. Scully M. L. Morley B. C. (2013). Associations between sports participation, adiposity, obesity-related health behaviors in Australian adolescents. Int. J. Behav. Nutr. Phys. Activity 10, 1–9. 10.1186/1479-5868-10-113 24088327 PMC3851783

[B62] Warburton P. Woods J. (1996). Observation of children's physical activity levels during primary school physical education lessons. Eur. J. Phys. Educ. 1, 56–65. 10.1080/1740898960010106

[B63] Wickel E. E. Eisenmann J. C. (2007). Contribution of youth sport to total daily physical activity among 6- to 12-yr-old boys. Med. Sci. Sports Exerc. 39, 1493–1500. 10.1249/mss.0b013e318093f56a 17805079

